# Effect of maternal and post-weaning dietary chitosan oligosaccharide supplementation on reproductive performance of sow and gut growth and development in offspring

**DOI:** 10.3389/fvets.2025.1666462

**Published:** 2025-09-23

**Authors:** Tingting Fang, Ying Yao, Gang Tian, Ping Zheng, Jie Yu, Hui Yan, Junning Pu, Bing Yu

**Affiliations:** Key Laboratory of Animal Disease-Resistance Nutrition, Animal Nutrition Institute, Sichuan Agricultural University, Chengdu, China

**Keywords:** chitosan oligosaccharide, sow, piglet, intestine, growth

## Abstract

This study investigated the effects of dietary chitosan oligosaccharide (COS) in sows on the reproductive performance and milk composition of sows, as well as the intestinal growth and development of piglets. A total of forty pregnant sows (average parity = 4) at day 90 of gestation were randomly divided into two groups and fed a basal diet (CONm, *n* = 20) or a diet supplemented with 100 mg/kg COS (COSm, *n* = 20) from late gestation to lactation. At 24 days of age, a 2 × 2 factorial treatment arrangement was employed with two independent factors: the sow diet and the piglet diet. The piglets selected from the same litter of sows fed CONm or COSm were assigned to receive either a control diet (CONp, *n* = 6) or a diet containing 100 mg/kg COS (COSp, *n* = 6) for 14 days. Maternal COS supplementation shortened the weaning-to-estrus interval and improved milk composition, up-regulated jejunal mRNA expressions of *CLDN3*, *SDCBP2*, *LDHA*, *MAP2K1*, and *PCK1*, ileal concentrations of IL-10 and IFN-γ in 2-days old suckling piglets (*p* < 0.05); and enhanced plasma TG level, jejunal mRNA expressions of *CLDN7* and *HNF4A*, ileal concentrations of IL-10 and *CD163* mRNA expressions in 24-days old suckling piglets (*p* < 0.05). In 38-days old weaning piglets, maternal COS enhanced the ratio of *Lactobacillus* spp.*-to-Escherichia coli*, butyrate, and propionate concentrations in cecal digesta, as well as decreased the concentrations of IL-10 and IFN-γ in ileal mucosa (*p* < 0.05), and the concentration of *Escherichia coli* (*p* < 0.05) in cecal digesta. Meanwhile, COS supplementation in weaned piglets increased the jejunal mRNA level of *CD163*, and cecal acetate, propionate, and total SCFA contents (*p* < 0.05). There were interaction effects between dietary COS in sow and dietary COS in piglet on ileal density, jejunal crypt depth and jejunal *mTOR* expression (*p* < 0.05). These results indicated that maternal COS intervention can enhance the reproductive performance and milk composition in sows, while improving intestinal growth and immunity parameters of suckling piglets. Furthermore, supplementing both sows and piglets with COS appeared to be a more effective way to promote gut growth in weaning piglets.

## 1 Introduction

Intestine fulfills a variety of functions, including traditional nutrient digestion and absorption, as well as certain endocrine and immunological functions (1). Intestinal functionality is mainly dependent on its organogenesis and maturation, while ontogenetic development of intestine is a highly organized process, which is critical for mammalian health and survival. The rapid fetal intestine growth commonly occurs during late gestation in preparation for the transition from placental nutrition before birth to fully enteral nutrition in postnatal life (2). In addition, the intestinal development not only begins during fetal life but also continues after birth with rapid organs and function maturation. A wide range of maternal and fetal factors are associated with ensuring normal intestinal organogenesis, while nutritional modifications during prenatal and postnatal periods play a crucial role in the structural and functional development (3). Variation in maternal nutrient intake during gestation or infant nutrient intake early in life can influence intestinal development and have long-term effects on health and performance (4).

Chitosan oligosaccharide (COS) is an oligosaccharide composed of *β*-1-4 linked D-glucosamine and N-acetyl-D-glucosamine units, obtained through chemical, enzymatic or radiation degradation of chitosan (5). Because of its water solubility, non-cytotoxic, excellent biocompatibility, and high intestinal adsorption properties, the potential application of COS as a feed additive has received considerable interest (6). Current studies indicated that maternal COS intervention enhanced sow health status by enhancing antioxidant defenses and improving the intrauterine environment for fetal development (7, 8). Additionally, COS upregulated transplacental amino acid transporters, thereby promoting maternal-fetal nutrient transfer critical for fetal development (9). These effects collectively elevated neonatal piglet survival rates, thereby increasing overall reproductive performance. However, the specific pathways through which COS enhances reproductive efficiency in sows are still unknown. Furthermore, dietary supplementation of COS during gestation and lactation also promoted the morphological development of small intestine, and improved the intestinal antioxidant capacity and immunity in suckling piglets (7, 10). However, the long-term benefits of maternal COS supplementation on intestinal development and health status in weaned piglets after remains unexplored.

Another challenge to postnatal gut growth and development in pigs is the process of weaning, which involves significant changes in physiology, microbiology and immunology. Previous studies have shown that supplementing piglets’ diets with COS can reduce weaning stress by improving intestinal structure integrity, barrier function, and immune response during early weaning (11–13). Additionally, dietary COS supplementation has been shown to modulate the microbiology changes by increasing beneficial bacteria and reducing pathogenic bacteria counts, thereby enhancing the growth performance of the pigs (11, 12). However, the synergistic benefits of combined maternal and post-weaning COS administration remain unexplored. Therefore, we hypothesized that maternal COS supplementation in sows and continued COS supplementation after weaning in piglets would exert beneficial effects on intestinal health of piglets by improving intestinal growth and development. Therefore, the aim of this study was to explore the effects of maternal dietary COS supplementation during late gestation and lactation, as well as continued dietary COS intervention in piglet after weaning on growth performance, intestinal morphology, immune function, and intestinal microbiota of piglets.

## 2 Materials and methods

The experimental procedures used in this study were approved by the Animal Care and Use Committee of Sichuan Agricultural University (China) and performed in accordance with the guidelines of the National Research Council’s Guide for the Care and Use of Laboratory Animals (No. SYXK (Sichuan) 2019–187).

### 2.1 Animals and experimental design

In this study, a total of 40 pregnant sows (Large White × Landrace) with the average parity of 4 at day 90 of gestation were obtained from a commercial pig farm (Sichuan, China). All sows were assigned to two groups: Control group (CONm, basal diet, *n* = 20), and COS group (COSm, basal diet supplemented with 100 mg/kg COS, *n* = 20). The basal diets were formulated to meet the nutrient requirements of gestating sows or lactation sows, respectively, as recommended by the National Research Council (NRC, 2012). The compositions and nutrients levels were shown in Tables 1, 2.

**Table 1:** Composition and nutrient levels of the basal diet for sow during late gestation (as fed-basis).

Ingredient	Content, %	Nutrient levels^1^	Content
Corn	42.3	Dry Matter, %	87.58
Barley	21.0	Crude protein, %	14.24
Soybean meal	13.6	ME, Mcal/kg	3.19
Wheat bran	16.0	Ca, %	1.21
Soybean oil	1.80	Available P, %	0.46
Limestone	2.00	SID-Lys, %	0.56
Dicalcium phosphate	1.60		
L-Lysine∙HCl	0.08		
Betaine	0.07		
Choline chloride	0.15		
Sodium chloride	0.40		
Potassium chloride	0.50		
Mineral and vitamin premix^2^	0.50		
Total	100		

^1^Nutrient levels are calculated according to the composition of the basal diet. ME, metabolic energy; SID, standard ileal digestibility.

^2^Premix supplied the following per kg of diet: copper, 10 mg as copper sulfate; iron, 80 mg as iron sulfate; zinc, 100 mg as zinc sulfate; manganese, 25 mg as manganese sulfate; selenium, 0.15 mg as sodium selenite; iodine, 0.14 mg as potassium iodide, vitamin A, 4000 IU; vitamin D3, 800 IU; vitamin E, 441 IU; menadione, 0.5 mg; thiamine, 1.0 mg; riboflavin, 3.75 mg; vitamin B6, 1.0 mg; vitamin B12, 15 μg niacin, 10 mg; pantothenic acid, 12 mg; folic acid, 1.3 mg; D-biotin, 200 μg.

**Table 2:** Composition and nutrient levels of the basal diet for sow during lactation (as fed-basis).

Ingredient	Content, %	Nutrient levels^1^	Content
Corn	42.4	Dry Matter, %	87.74
Barley	19.0	Crude protein, %	17.24
Soybean meal	19.5	ME, Mcal/kg	3.11
Extruded soybean	6.00	Ca, %	0.89
Fish meal	2.00	Available P, %	0.36
Wheat bran	4.00	SID-Lys, %	0.98
Soybean oil	2.80	SID-Met, %	0.26
Limestone	1.30	SID-Trp, %	0.22
Dicalcium phosphate	1.20	SID-Thr, %	0.45
L-Lysine∙HCl	0.26	SID-Val, %	0.74
DL-Met	0.04		
L-Trp	0.01		
L-Thr	0.09		
Val	0.05		
Betaine	0.06		
Choline chloride	0.15		
Sodium chloride	0.40		
Potassium chloride	0.20		
Mineral and vitamin premix^2^	0.54		
Total	100		

^1^Nutrient levels are calculated according to the composition of the basal diet. ME, metabolic energy; SID, standard ileal digestibility.

^2^Premix supplied the following per kg of diet: copper, 20 mg as copper sulfate; iron, 80 mg as iron sulfate; zinc, 100 mg as zinc sulfate; manganese, 25 mg as manganese sulfate; selenium, 0.15 mg as sodium selenite; iodine, 0.14 mg as potassium iodide, vitamin A, 4000 IU; vitamin D3, 800 IU; vitamin E, 441 IU; menadione, 0.5 mg; thiamine, 1.0 mg; riboflavin, 3.75 mg; vitamin B6, 1.0 mg; vitamin B12, 15 μg niacin, 10 mg; pantothenic acid, 12 mg; folic acid, 1.3 mg; D-biotin, 200 μg.

Nutrient levels in the experimental diet were measured according to procedures outlined by the Association of Official Analytical Chemists (2007) for the crude protein (method 976.05). The calcium (Ca) (GB/T 6436–2018) and phosphorus (P) (GB/T 6437–2018) levels were analyzed according to China National Standard, and for Lys using ninhydrin for postcolumn derivatization by an Amino Acid Analyzer (Hitachi High Technologies America Inc., Pleasanton, CA, USA), respectively. The metabolic energy was calculated according to the NRC (2012). The sows were provided with 3 kg of gestation diet daily (8,00 and 14,00) from day 91 of gestation until parturition. Following delivery, sows were fed with lactation diet and increased gradually until the sows could feed ad libitum (8,00, 14,00, and 21,00) from day 1 post-farrowing to day 24. Within the first 48 h after farrowing, the piglets were weighed and then the litter sizes were adjusted to 12 ± 1 piglets per litter by cross-fostering in the same treatment.

All piglets were individually weighed and weaned at 24 days post-birth. A 2 × 2 factorial treatment arrangement was employed with two fixed factors: Sow diet (CONm (basal diet) or COSm (basal diet + 100 mg/kg COS)) and piglet diet (CONp (basal diet) or COSp (basal diet + 100 mg/kg COS)). Within each sow treatment (CONm/COSm), piglets from multiple litters were assigned to piglet diets (CONp/COSp) based on body weight and gender. The piglets from multiple assigned litters were mixed to form replicate pens for each treatment combination, ensuring that littermates were distributed to different pens within their dietary treatment where possible. Thus, there were four piglet treatments: CONm-CONp, CONm-COSp, COSm-CONp and COSm-COSp. Each treatment consisting five replicates with 15 piglets per replicate, with sex strictly balanced such as half male and half female where possible. The piglet basal diet was formulated based on NRC 2012 recommendations (Table 3). During the 14-day period, the piglets had free access to feed and water. Feed intake of each replicate was recorded daily, and the body weight (BW) of each piglet was measured at the end of the experiment to calculate the average daily feed intake (ADFI), average daily gain (ADG), and the gain-to-feed ratio.

**Table 3:** Composition and nutrient levels of the basal diet for weaning piglets (as fed-basis).

Ingredient	Content, %	Nutrient levels^1^	Content
Corn	39.0	Dry Matter, %	87.73
Borken rice	20.5	Crude protein, %	18.09
Soybean meal	9.26	ME, Mcal/kg	3.29
Fermented soybean meal	7.50	Ca, %	0.74
Whey power	5.00	Available P, %	0.36
Extruded soybean	10.0	SID-Lys, %	1.47
Fish meal	3.50	SID-Met, %	0.37
Glucose	2.00	SID-Trp, %	0.26
Soybean oil	0.60	SID-Thr, %	0.45
Limestone	0.45		
Monocalcium phosphate	0.40		
L-Lysine∙HCl	0.47		
DL-Met	0.05		
L-Trp	0.05		
L-Thr	0.16		
Choline chloride	0.12		
Sodium chloride	0.40		
Mineral and vitamin premix^2^	0.54		
Total	100		

^1^Nutrient levels are calculated according to the composition of the basal diet. ME: metabolic energy, SID: standard ileal digestibility.

^2^Premix supplied the following per kg of diet: Zn (ZnSO4. H2O), 100 mg; Mn (MnSO4. H2O), 10 mg; Fe (FeSO4. H2O), 100 mg; Cu (CuSO4.5H2O), 10 mg; Se (Na2SeO3), 0.35 mg and I (KI), 0.28 mg. vitamin A, 11,000 IU; vitamin D3, 800 IU; vitamin E, 44 IU; niacin, 55 mg; pantothenic acid, 33 mg; riboflavin, 10 mg; vitamin K, 2.4 mg and vitamin B12, 44 μg.

Chitosan oligosaccharide used in this study had a deacetylation degree of approximately 95% and an average molecular weight below 1,000 Da with maltodextrin is used as a carrier, it was provided by Zhongke Rongxin Biotechnology Co., Ltd. (Suzhou, China), with dosage based on prior research in sows (14).

### 2.2 Sample collection

After parturition, the total and average birth weight of piglets were recorded prior to nursing (1 sow/pen, *n* = 20). After injecting oxytocin (1 mg) intravenously, colostrum samples (2 h post-farrowing) and milk samples (day 7 of lactation) were collected manually from 8 sows with similar body weight, parity, and backfat thickness per diet, and stored at −20 °C until analysis.

During lactation, six male piglets from each group (per piglet from per sow) were bled on d 2 and d 24 of age, respectively. Blood samples of piglets were collected using vacuum blood collection tube with EDTA from the jugular vein. The samples were then centrifuged at 3000 × g for 10 min at 4 °C, and the plasma was subsequently collected and stored at −20 °C for later analysis.

Additionally, six male piglets with average BW per treatment were used for intestinal sampling on d 2, d 24 and d 38, respectively. The piglets were euthanized via jugular puncture under anesthesia with an intravenous injection of a 4% sodium pentobarbital solution. Mucosal scrapings of jejunum and ileum were rapidly snap-frozen and stored at −80 °C for further analysis. Furthermore, on d 38, the entire small intestines were immediately removed from the abdomen of each piglet, removed digesta in the intestine with physiological saline, weighed and measured for the length of jejunum and ileum. Jejunal tissues samples were fixed in a 4% paraformaldehyde solution for morphological analysis. Cecum contents were quickly collected and stored at −80 °C for future analysis of gut microflora and short-chain fatty acids (SCFA).

### 2.3 Determination of the reproductive performance of sows

Reproductive performance of each sow was assessed by monitoring the number of piglets born (total, alive and stillborn) and the number of piglets with a birth weight less than 1,000 g. Individual piglet weights at birth and weaning were recorded. Daily feed intake during lactation was recorded for each sow. The weaning-to-estrus interval from weaning to estrus was also recorded.

### 2.4 Determination of colostrum and milk composition

The fat, protein, lactose, and solids-not-fat content of the colostrum and milk samples were measured with an automated milk analyzer (Milk-Yway-CP2; Zhejiang, China), with results reported as percentage. The concentrations of transforming growth factor -β1 (TGF-β1) in colostrum and milk were determined using a specific assay kit (Beijing 4A Biotech Co., Ltd., Beijing, China) according to the manufacturer’s instructions.

### 2.5 Plasma parameter measurements in suckling piglets

The commercial available assay kits were used for ELISA measurements of plasma fructose and immunoglobulin A (IgA) concentrations (Nanjing Jiancheng Bioengineering Institute, Nanjing, China) according to the manufacturer’s instructions. Plasma concentrations of triglyceride (TG) and immunoglobulin G (IgG) were determined using corresponding commercial kits (Sichuan Maker Biotechnology Inc., Chengdu, China) with an automatic biochemical analyzer (Hitachi 7,020, Hitachi High Technologies Corporation, Tokyo, Japan).

### 2.6 Morphological analysis

The densities of jejunum and ileum were calculated using the formula: intestinal density (g/m) = intestinal weight/intestinal length. The jejunal samples were fixed in paraformaldehyde solution, rinsed with physiological saline, and then embedded in paraffin wax. Sections were dewaxed, hydrated, and then stained with hematoxylin and eosin for morphology determination. Ten well-orientated and intact villi and crypts were measured with an image processing and analysis system (Image-Pro Plus 6.0, Media Cybernetics, USA), and the mean villus height and crypt depth, as well as the villus height-to-crypt depth ratio were calculated. The goblet cell counts per villus were also assessed.

### 2.7 Ileal cytokine content determinations

Ileal mucosal were homogenized in ice-cold physiological saline (1:9, w/v) and then centrifuged at 3000 × g for 15 min at 4 °C. The supernatants were used to measure the interleukin-10 (IL-10), interferon-γ (IFN-*γ*), and secretory immunoglobulin A (sIgA) levels according to the manufacturer’s instructions by using the respective ELISA kits (Beijing 4A Biotech Co., Ltd., Beijing, China).

### 2.8 RNA extraction and real-time PCR assay

Gene transcriptional abundances of intestine were detected using reverse transcription polymerase chain reaction (RT-PCR). Briefly, total RNA was extracted with Trizol reagent and reverse-transcribed into cDNA using the Prime-Script® RT Master Mix kit (Takara Bio Inc., Dalian, China) following the manufacturer’s instructions. Real-time PCR assays were performed with SYBR® green system (Takara Bio Inc., Dalian, China) on complementary DNA samples using a QuanStudio 6 Flex Real-Time PCR System (Applied Biosystems, Foster City, CA, USA). The most stable housekeeping genes (*HPRT1, PPIA,* and *TBP*) were chosen for normalization, and the relative mRNA abundance of the analyzed genes was calculated using the 2^−ΔΔCT^ method (15). Primer sequences for individual genes were listed in Supplementary Table S1.

### 2.9 Cecal *Lactobacillus* spp., and *Escherichia coli* measurements

Total DNA was extracted from caecum digesta samples using the EZNA® Stool DNA kit (Omega Bio-Tek, Doraville, CA, USA) following the manufacturer’s instructions. Quantitative RT-PCR for *Total bacteria*, *Lactobacillus* spp. and *Escherichia coli* was performed with fluorescent oligonucleotide probes using the commercial available SuperReal Premix Kit (Tiangen Biotech, Ltd., Beijing, China). Amplification was performed using a CFX 96 System (Bio-Rad Laboratories, Inc., Hercules, CA, USA). Primer sequence data for *Total bacteria* forward primer (5′-3′) was ACTCCTACGGGAGGCAGCAG, reverse primer (3′-5′) was ATTACCGCGGCTGCTGG, *Lactobacillus* spp. forward primer (5′-3′) was GAGGCAGCAGTAGGGAATCTTC, reverse primer (3′-5′) was CAACAGTTACTCTGACACCCGTTCTTC, and for *Escherichia coli* forward primer (5′-3′) was CATGCCGCGTGTATGAAGAA, reverse primer (3′-5′) was CGGGTAACGTCAATGAGCAAA. The results are expressed as the percentage of *Lactobacillus* and *Escherichia coli* within the total bacterial population.

### 2.10 Short chain fatty acid analysis

Extraction of SCFA, including acetate, propionate and butyrate, from cecum digesta by organic solvent was performed as described in previous study (16). Cecum digesta (approximately 1 g) was mixed with deionized water and allowed to stand for 30 min at 4 °C before being centrifuged (1,000 × g, 4 °C) for 10 min. The supernatant was then mixed with 0.24 mL of 25% metaphosphate and centrifuged again (1,000 × g, 4 °C) for another 10 min to obtain the final supernatant, which was subsequently prepared for gas chromatography analysis (Varian, Inc., Artaud, CA, USA).

### 2.11 Statistical analysis

The data on reproductive performance and milk composition in sows, plasma parameters, jejunal relative mRNA expression, and ileal immunity parameters in suckling piglets (day 2 and day 24 of age) were analyzed using a t-test within SPSS 22.0 (IBM SPSS Company, Chicago, IL, USA). The growth performance, intestinal morphology, jejunal relative mRNA expression, ileal immunological parameters, and cecal microbes and SCFA productions in weaning piglets (d 38 of age) were analyzed as a completely randomized design with a 2 × 2 factorial treatment arrangement by ANOVA using the GLM procedure of SPSS, and Tukey multiple comparisons were performed when interactions were significant. The statistical model included the main effects of sow dietary COS treatment (CONm or COSm), piglet dietary COS treatment (CONp or COSp), and their interactions. The growth performance of weaning piglets (d 38 of age) was analyzed with the pen as the statistical unit, while all other parameters were evaluated using individual sows/piglets as the experimental unit. All results obtained in this experiment were expressed as means and the SEM. Differences were considered as statistically significant when *p* < 0.05. The 0.05 ≤ *p* ≤ 0.1 was considered a trend.

## 3 Results

### 3.1 Reproductive performance

As shown in Table 4, maternal COS supplementation decreased the weaning-to-estrus interval in sows (*p* < 0.05) and tended to increase the number of piglets with a birth weight greater than 1.0 kg (*p* = 0.081) and the piglet ADG before weaning (*p* = 0.100). However, there was no effect on the numbers of total born, born alive, and stillborn piglets and mean piglet weight at birth due to maternal COS supplementation, and no treatment effect was observed for sows’ ADFI during lactation.

**Table 4:** Effect of chitosan oligosaccharide (COS) in the maternal diet on sow reproductive performance.^1^

Item	Treatment		*p*-value
	CONm	COSm	
Total number of born, *n*	16.4 ± 0.48	16.0 ± 0.40	0.662
Number of born alive, *n*	14.7 ± 0.51	14.4 ± 0.46	0.715
Number of stillborn piglet, *n*	1.67 ± 0.04	1.59 ± 0.04	0.937
Piglet mean BW at birth, kg	1.23 ± 0.03	1.31 ± 0.04	0.275
Number of piglets > 1.0 kg, n	11.8 ± 0.32	12.4 ± 0.28	0.081
Piglet mean BW at weaning, kg	6.93 ± 0.13	7.36 ± 0.20	0.101
Piglet ADG during suckling, g/d	239 ± 4.28	253 ± 3.76	0.100
Sows’ ADFI during lactation, kg	5.89 ± 0.21	5.98 ± 0.21	0.681
Weaning-to-estrus interval, d	3.94 ± 0.25	3.33 ± 0.19	0.023

^1^All values are expressed as the means ± SEM, *n* = 20. CONm: sows fed control diet during late gestation and lactation; COSm: sows fed control diet containing 100 mg/kg COS during late gestation and lactation; BW, body weight; ADG, average daily gain; ADFI, average daily feed intake.

### 3.2 Colostrum and milk composition

According to Table 5, there were no differences in the concentrations of fat, protein, lactose, solids-not-fat, and TGF-β1 in colostrum between CONm sows and COSm sows. Maternal COS supplementation increased (*p* < 0.05) the milk contents of protein, lactose, solids-not-fat. The TGF-β1 tended to be higher (*p* = 0.053) in COSm group compared to CONm group.

**Table 5:** Effect of chitosan oligosaccharide (COS) in the maternal diet on colostrum and milk composition.^1^

Item	Treatment		*p*-value
	CONm	COSm	
Colostrum			
Fat, %	4.48 ± 0.22	3.99 ± 0.40	0.171
Protein, %	6.92 ± 0.32	7.41 ± 0.50	0.284
Lactose, %	10.1 ± 0.48	10.9 ± 0.76	0.289
Solids-not-fat, %	18.8 ± 0.88	20.1 ± 1.37	0.292
TGF-β1, pg./mL	277 ± 70.3	390 ± 95.8	0.136
Milk at day 7 of lactation			
Fat, %	7.05 ± 0.44	7.09 ± 0.48	0.920
Protein, %	3.78 ± 0.15	3.94 ± 0.06	0.027
Lactose, %	5.36 ± 0.21	5.61 ± 0.08	0.011
Solids-not-fat, %	10.1 ± 0.16	10.5 ± 0.16	0.029
TGF-β1, pg./mL	231 ± 2.45	320 ± 5.26	0.053

^1^All values are expressed as the means ± SEM, *n* = 8. CONm: sows fed control diet during late gestation and lactation; COSm: sows fed control diet containing 100 mg/kg COS during late gestation and lactation; TGF-β1, transforming growth factor -β1.

### 3.3 Plasma fructose, TG, IgG and IgA contents in nursing piglets

**Figure 1:**
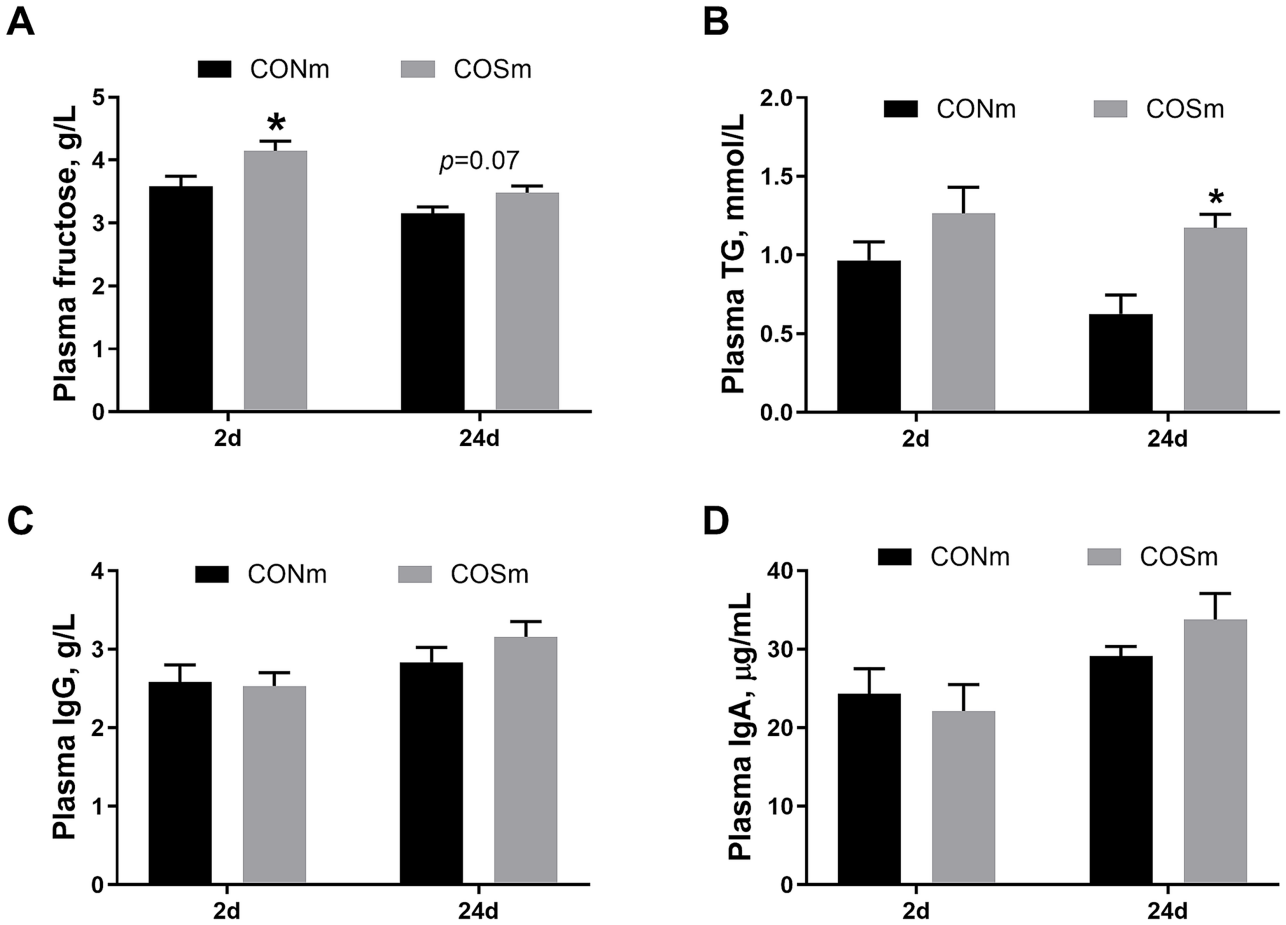
Effects of dietary COS supplementation in sows during late gestation and lactation on plasma parameters in suckling piglets (d 2 and d 24 of age). **(A)** Plasma concentrations of fructose; **(B)** Plasma concentrations of triglyceride; **(C)** Plasma concentrations of immunoglobulin A; **(D)** Plasma concentrations of immunoglobulin G. COS: Sows were fed with basal diet containing 100 mg/kg COS. All values are expressed as the means ± SEM, *n* = 6. * Means a significant difference on the vertical bar (*p* < 0.05).

As shown in Figure 1, compared with nursing piglets from CONm sows, nursing piglets from COSm sows exhibited higher concentrations of plasma fructose at 2 days of age (*p* < 0.05, Figure 1A), as well as tended to elevate concentrations of fructose (*p* = 0.072) and TG (*p* < 0.05, Figure 1B) in plasma at 24 days of age. However, the contents of plasma IgG (Figure 1C) and IgA (Figure 1D) in nursing piglets were not affected by dietary COS supplementation in sows.

### 3.4 Gene expression of jejunal in nursing piglets

**Figure 2:**
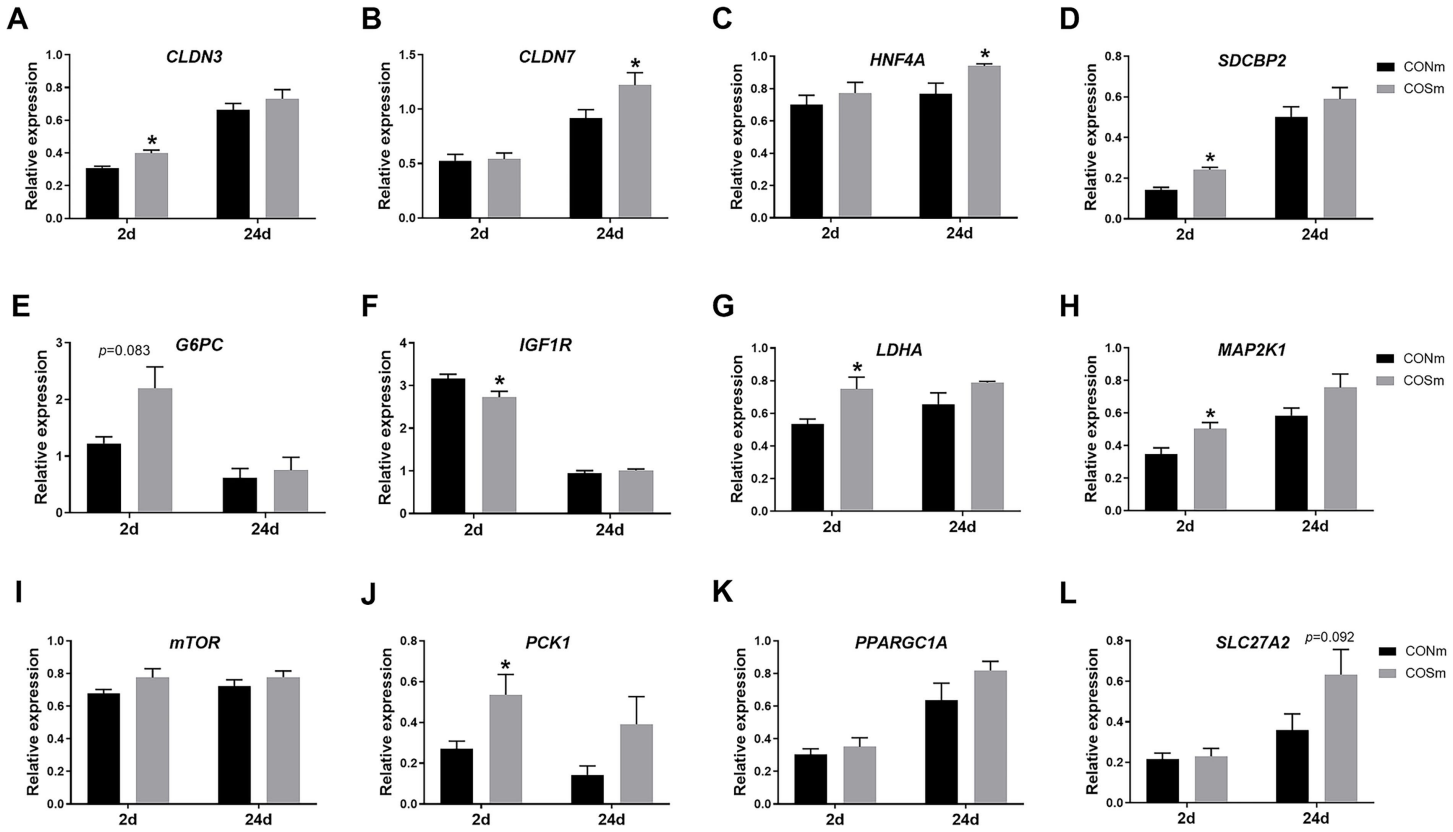
Effects of dietary COS supplementation in sows during late gestation and lactation on jejunal relative mRNA expression in suckling piglets (d 2 and d 24 of age). **(A)** CLDN3, Claudin-3; **(B)** CLDN7, Claudin-7; **(C)** HNF4A, Hepatocyte nuclear factor 4 alpha; **(D)** SDCBP2, Syndecan binding protein 2; **(E)** G6PC, Glucose 6 phosphatase catalytic subunit; **(F)** IGF1R, Insulin like growth factor 1 receptor; **(G)** LDHA, Lactate dehydrogenase A; **(H)** MAP2K1, Mitogen activated protein kinase kinase 1; **(I)** mTOR, Mammalian target of rapamycin; **(J)** PCK1, Phosphoenolpyruvate carboxykinase 1; **(K)** PPARGC1A, Peroxisome proliferator-activated receptor gamma, coactivator 1 alpha; **(L)** SLC27A2, Solute carrier family 27 member 2. COS: Sows were fed with basal diet containing 100 mg/kg COS. All values are expressed as the means ± SEM, *n* = 6. * Means a significant difference on the vertical bar (*p* < 0.05).

Supplementation of COS in sow diet resulted in increased jejunal mRNA levels of *CLDN3* (*p* < 0.05, Figure 2A), *SDCBP2* (*p* < 0.05, Figure 2D), *G6PC* (*p* = 0.083, Figure 2E), *LDHA* (*p* < 0.05, Figure 2G), *MAP2K1* (*p* < 0.05, Figure 2H) and *PCK1* (*p* < 0.05, Figure 2J) but decreased the mRNA level of *IGF1R* (*p* < 0.05, Figure 2F) in 2 days of age nursing piglets. The mRNA levels of jejunal *CLDN7* (*p* < 0.05, Figure 2B) and *HNF4A* (*p* < 0.05, Figure 2C) were increased, and mRNA level of *SLC27A2* (*p* = 0.092, Figure 2L) tended to increase in nursing piglets from COS sows at 24 days of age.

### 3.5 Ileal immunological parameters in nursing piglets

**Figure 3:**
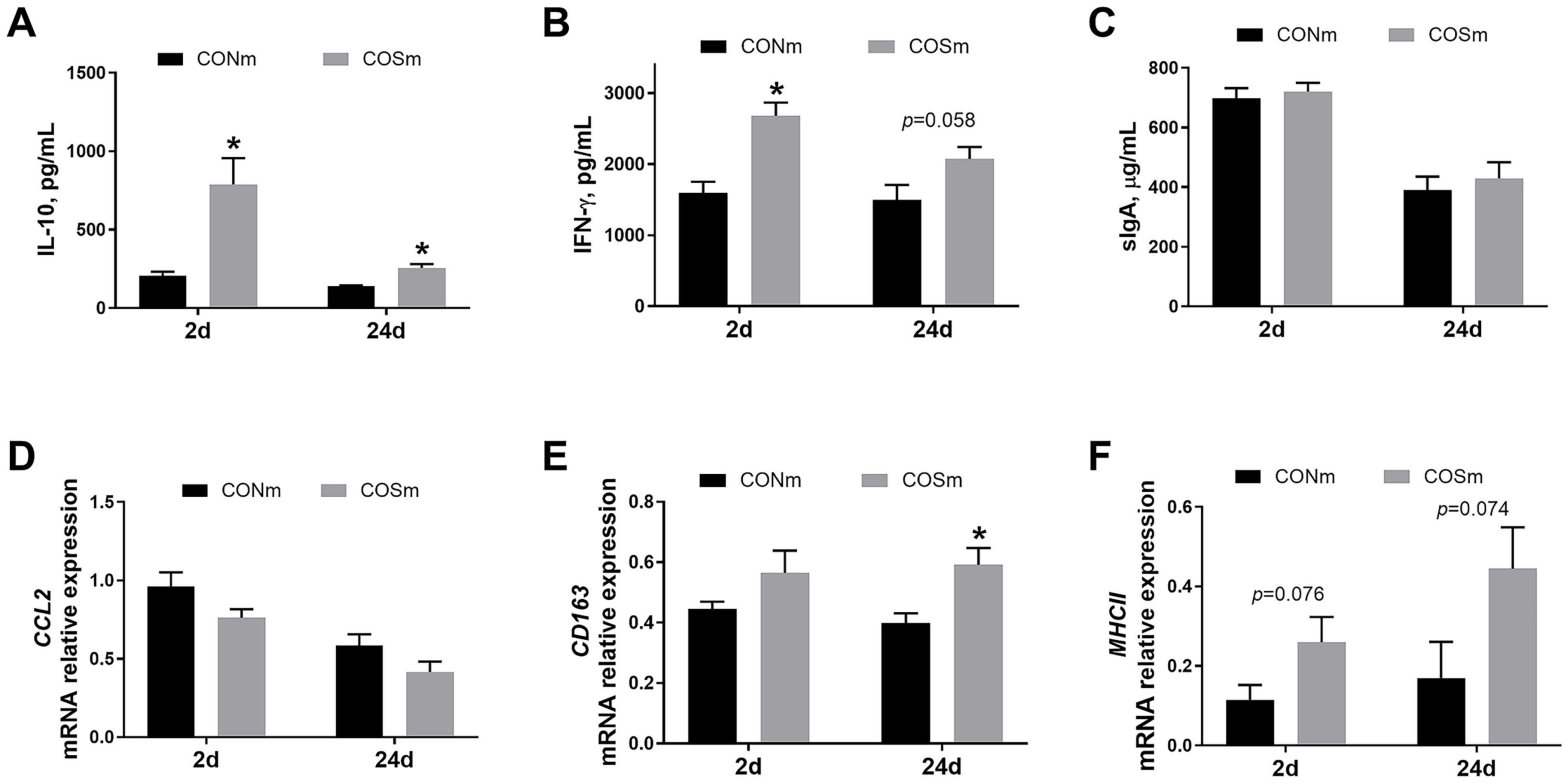
Effects of dietary COS supplementation in sows during late gestation and lactation on ileal immune response in suckling piglets (d 2 and d 24 of age). **(A)** IL-10, Interleukin-10; **(B)** IFN-γ, Interferon-γ; **(C)** sIgA, Secretory immunoglobulin A; **(D)** CCL2, Chemokine ligand 2, c-c motif; **(E)** CD163, Cluster of differentiation 163; **(F)** MHCII, Major histocompatibility complex class 2. COS: Sows were fed with basal diet containing 100 mg/kg COS. All values are expressed as the means ± SEM, *n* = 6. *Means a significant difference on the vertical bar (*p* < 0.05).

As shown in Figure 3, elevated IL-10 and IFN-γ (*p* < 0.05) levels were observed in the ileum of suckling piglets from COSm sows compared to CONm sows at 2 days of age, and the ileal IL-10 (*p* < 0.05) and IFN-γ (*p* = 0.058) levels were increased in piglets at age of 24 days from COSm sows. However, the levels of sIgA in ileal mucosa of piglets were not affected by COS diet in sow. Moreover, the ileal *MHCII* mRNA expression (*p* = 0.076) tended to increase in suckling piglets from COSm sows compared to CONm sows at 2 days of age, and the ileal mRNA levels of *CD163* (*p* < 0.05) and *MHCII* (*p* = 0.074) were increased in piglets at age of 24 days from COSm sows.

### 3.6 Growth performance in weaning piglets

**Figure 4:**
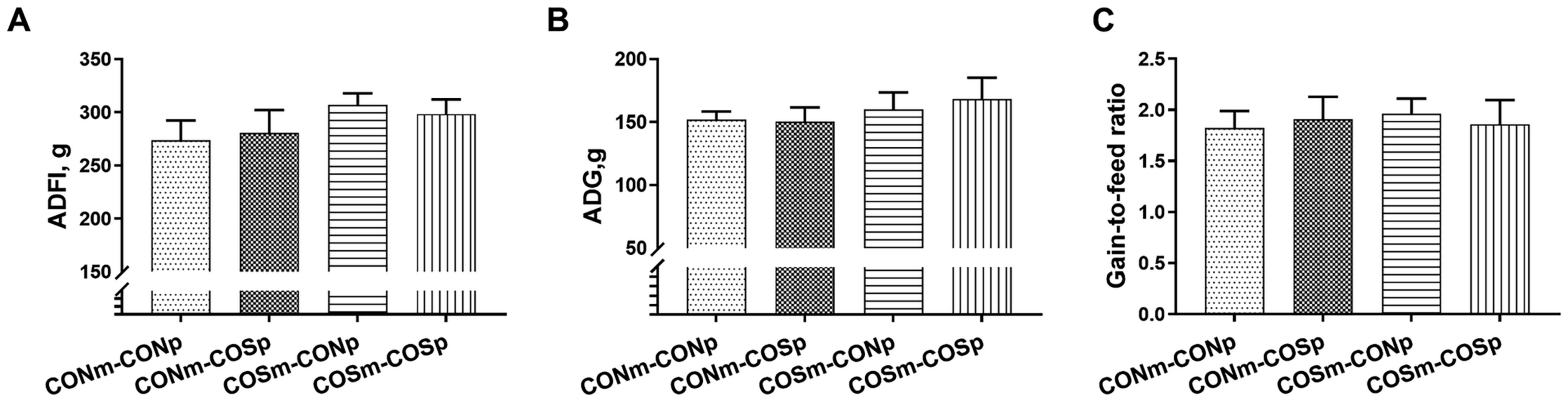
Effects of dietary COS supplementation on growth performance in weaning piglets (d 38 of age). **(A)** ADFI, Average daily feed intake; **(B)** ADG, Average daily gain; **(C)** Gain-to-feed ratio. CONm-CONp: Sows fed control diet during late gestation and lactation and piglets fed control diet after weaning. CONm-COSp: Sows fed control diet during late gestation and lactation and piglets fed control diet containing 100 mg/kg COS after weaning. COSm-CONp: Sows fed control diet containing 100 mg/kg COS during late gestation and lactation and piglets fed control diet after weaning. COSm-COSp: Sows fed control diet containing 100 mg/kg COS during late gestation and lactation and piglets fed control diet containing 100 mg/kg COS after weaning. All values are expressed as the means ± SEM, *n* = 6.

As shown in Figure 4, the supplementation of COS after weaning did not have a effect on piglet ADFI, ADG, and gain-to-feed ratio.

### 3.7 Intestinal morphology in weaning piglets

Based on Table 6, sows supplemented with COS improved the density of the jejunum and ileum in weaned piglets (*p* < 0.05). There was an interaction between sow dietary COS and piglet dietary COS on ileal density (*p* < 0.05) as well as jejunal crypt depth (*p* < 0.05), and there was a tendency of interaction between sow dietary COS and piglet dietary COS on jejunal villus height-to-crypt depth ratio (*p* = 0.057). The group supplemented with both maternal and piglet dietary COS had the highest ileal density compared to CONm-COSp and CONm-CONp groups (*p* < 0.05). The jejunal crypt depth of piglets from the groups of CONm-COSp and COSm-CONp were lower than that of CONm-CONp group (*p* < 0.05). No differences were observed in the goblet cells and villus height between different treatments in weaning piglets.

**Table 6:** Effects of dietary chitosan oligosaccharide (COS) supplementation on intestinal morphology analysis in weaning piglets (day 38 of age).^1^

Item	CONm		COSm		SEM	*p*-value		
	CONp	COSp	CONp	COSp		m	p	m × p
Jejunal density (g/m)	261.25	280.30	349.67	357.00	12.475	< 0.001	0.487	0.757
Ileal density (g/m)	280.50^a^	327.92^ab^	390.88^b^	355.50^b^	12.397	0.003	0.765	0.032
Jejunal villus height (μm)	407.24	397.69	380.88	406.34	7.116	0.549	0.590	0.242
Jejunal crypt depth (μm)	111.11^b^	85.44^a^	84.25^a^	90.93^ab^	3.725	0.109	0.152	0.019
Jejunal villus height to crypt depth ratio	3.68	5.00	4.62	4.48	0.197	0.566	0.118	0.057
Goblet cell (n/villous)	8.95	11.57	12.14	11.97	0.722	0.225	0.402	0.343

^1^All values are expressed as the means and SEM, *n* = 6. CONm: sows fed control diet during late gestation and lactation. COSm: sows fed control diet containing 100 mg/kg COS during late gestation and lactation. CONp: piglets fed control diet after weaning. COSp: piglets fed control diet containing 100 mg/kg COS after weaning. m: main effect of sow dietary COS treatment; p: main effect of piglet dietary COS treatment; m × p: the interactions of main effects of sow dietary COS treatment and piglet dietary COS treatment.

^a,b^Means a significant difference within an item (*p* < 0.05).

### 3.8 Gene expression of jejunal in weaning piglets

As shown in Table 7, COS supplementation in sows increased the mRNA level of *PPARGC1A* (*p* < 0.05), and tended to evaluate mRNA levels of *LDHA* (*p* = 0.053) and *CD163* (*p* = 0.084), while decreased the mRNA levels of *CCL2* (*p* = 0.094) with tendency in weaned piglets at d 38 of age. In weaned piglets, COS supplementation in nursery diets increased the mRNA levels of *IGF1R* (*p* = 0.074) and *CD163* and *HNF4A* (*p* < 0.05), but decreased the mRNA level of *MHCII* (*p* = 0.002). An interaction between sow dietary COS and piglet dietary COS was observed for jejunal mTOR expression (*p* < 0.05). The mRNA levels of *CLDN3, CLDN7, SDCBP2, G6PC, MAP2K1, PCK1, TGFB2* and *SLC27A2* were not affected by dietary COS supplementation in either sows or piglets.

**Table 7:** Effect of dietary chitosan oligosaccharide (COS) supplementation on intestinal gene expression of weaned piglets (day 38 of age).^1^

Item^2^		CONm		COSm		SEM	*p*-value		
		CONp	COSp	CONp	COSp		m	p	m × p
Jejunum	CLDN3	1.00	1.14	1.01	1.01	0.036	0.442	0.342	0.355
	CLDN7	1.00	1.11	0.91	1.01	0.036	0.197	0.157	0.913
	HNF4A	1.00	1.15	0.98	1.11	0.033	0.613	0.042	0.815
	SDCBP2	1.00	1.01	0.98	0.98	0.041	0.767	0.960	0.921
	G6PC	1.00	0.83	0.84	0.78	0.100	0.619	0.605	0.799
	IGF1R	1.00	1.25	1.15	1.34	0.059	0.297	0.074	0.791
	LDHA	1.00	1.15	1.25	1.19	0.039	0.053	0.579	0.156
	MAP2K1	1.00	1.11	1.15	1.06	0.036	0.520	0.902	0.167
	mTOR	1.00^a^	1.23^b^	1.15^ab^	1.04^ab^	0.030	0.668	0.224	0.003
	PCK1	1.00	0.88	0.74	0.76	0.090	0.314	0.794	0.723
	PPARGC1A	1.00	1.19	1.44	1.43	0.053	< 0.001	0.303	0.215
	SLC27A2	1.00	1.30	1.23	1.26	0.066	0.491	0.209	0.320
Ileum	CCL2	1.00	0.90	0.83	0.83	0.037	0.094	0.532	0.580
	CD163	1.00	1.26	1.08	1.38	0.040	0.084	< 0.001	0.679
	MHCII	1.00	0.73	1.05	0.74	0.048	0.738	0.002	0.786
	TGFB2	1.00	1.09	1.05	1.21	0.051	0.407	0.236	0.745

^1^All values are expressed as the means and SEM, *n* = 6. CONm: sows fed control diet during late gestation and lactation. COSm: sows fed control diet containing 100 mg/kg COS during late gestation and lactation. CONp: piglets fed control diet after weaning. COSp: piglets fed control diet containing 100 mg/kg COS after weaning. m: main effect of sow dietary COS treatment; p: main effect of piglet dietary COS treatment; m × p: the interactions of main effects of sow dietary COS treatment and piglet dietary COS treatment.

^2^CLDN3, claudin-3; CLDN7, claudin-7; HNF4A, hepatocyte nuclear factor 4 alpha; SDCBP2, syndecan binding protein 2; G6PC, glucose 6 phosphatase catalytic subunit; IGF1R, insulin like growth factor 1 receptor; LDHA, lactate dehydrogenase A; MAP2K1, mitogen activated protein kinase kinase 1; mTOR, mammalian target of rapamycin; PCK1, phosphoenolpyruvate carboxykinase 1; PPARGC1A, peroxisome proliferator-activated receptor gamma, coactivator 1 alpha; SLC27A2, solute carrier family 27 member 2; CCL2, chemokine ligand 2, c-c motif; CD163, cluster of differentiation 163; MHCII, major histocompatibility complex class 2; TGFB2, transforming growth factor beta 2.

^a, b^ Means a significant difference within an item (*p* < 0.05).

### 3.9 Ileal immunological parameters in weaning piglets

The results of ileal immunological parameters in weaning piglets were presented in Table 8. There were no interactions between sow dietary COS and piglet dietary COS on ileal IL-10, IFN-γ, and sIgA. Meanwhile, piglets from COS-treated mothers exhibited lower concentrations of ileal IL-10 and IFN-γ compared to non-treated CONm piglets (*p* < 0.05).

**Table 8:** Effect of dietary chitosan oligosaccharide (COS) supplementation on ileal mucosa IL10, IFNγ and sIgA content of weaned piglets (day 38 of age).^1^

Item^2^	CONm		COSm		SEM	*p*-value		
	CONp	COSp	CONp	COSp		m	p	m × p
IL-10 (pg/mL)	200.22	176.65	168.26	158.52	6.440	0.049	0.179	0.570
IFN-γ (pg/mL)	1132.60	1104.95	814.03	797.29	50.571	0.001	0.791	0.948
sIgA (μg/mL)	429.93	392.82	407.90	422.08	13.185	0.897	0.682	0.363

^1^All values are expressed as the means and SEM, n = 6. CONm: sows fed control diet during late gestation and lactation. COSm: sows fed control diet containing 100 mg/kg COS during late gestation and lactation. CONp: piglets fed control diet after weaning. COSp: piglets fed control diet containing 100 mg/kg COS after weaning. m: main effect of sow dietary COS treatment; p: main effect of piglet dietary COS treatment; m × p: the interactions of main effects of sow dietary COS treatment and piglet dietary COS treatment.

^2^IL-10, interleukin-10; IFN-γ, interferon-γ; sIgA, secretory immunoglobulin A.

### 3.10 Cecal chyme *Lactobacillus* spp., *Escherichia coli*, and SCFA levels

The Table 9 presented the *Lactobacillus* spp., *Escherichia coli*, and SCFA levels in cecal chyme. Dietary supplementation of COS in sows increased the *Lactobacillus* spp.-to-*Escherichia coli* ratio (*p* < 0.05), butyrate (*p* < 0.05), and propionate (*p* < 0.05), as well as increased total SCFA in tendency (*p* = 0.071), while decreasing the concentration of *Escherichia coli* (*p* < 0.05) in the cecum. Chitosan oligosaccharide supplementation in piglets resulted in an increase (*p* < 0.05) in acetate, propionate and total SCFA. No interaction between sows and piglets supplemented COS was found among these cecal-related indexes.

**Table 9:** Effect of dietary chitosan oligosaccharide (COS) supplementation on cecal chyme *Lactobacillus* and *Escherichia coli* levels and SCFA content of weaned piglets (day 38 of age).^1^

Item	CONm		COSm		SEM	*p*-value		
	CONp	COSp	CONp	COSp		m	p	m × p
*Lactobacillus* (%)	67.82	65.88	67.25	68.56	1.182	0.679	0.901	0.522
*Escherichia coli* (%)	34.73	32.20	21.80	21.38	2.092	0.004	0.686	0.772
*Lactobacillus/ Escherichia coli*	1.85	1.95	3.25	3.33	0.185	< 0.001	0.699	0.973
Acetate (mmol/kg)	37.10	58.02	49.47	57.30	3.394	0.361	0.032	0.306
Propionate (mmol/kg)	13.43	16.75	19.29	24.84	1.315	0.004	0.049	0.603
Butyrate (mmol/kg)	5.05	8.32	9.63	10.25	0.729	0.019	0.143	0.311
Total SCFA	55.57	83.09	78.39	92.39	4.839	0.071	0.023	0.432

^1^All values are expressed as the means and SEM, *n* = 6. CONm: sows fed control diet during late gestation and lactation. COSm: sows fed control diet containing 100 mg/kg COS during late gestation and lactation. CONp: piglets fed control diet after weaning. COSp: piglets fed control diet containing 100 mg/kg COS after weaning. m: main effect of sow dietary COS treatment; p: main effect of piglet dietary COS treatment; m × p: the interactions of main effects of sow dietary COS treatment and piglet dietary COS treatment.

## 4 Discussion

Nutritional feed supplements and additives are commonly used to improve health status, reproductive and growth performances of intensively raised farm swine. In this study, there were no significant effects observed on the number of total born piglets and birth weight when COS was supplemented during late gestation, likely due to factors such as fertilization rate and early prenatal mortality being less influenced by dietary supplements shortly before parturition (17). Similar results were reported in a previous study that sow dietary COS enhanced litter size and birth weight when supplementation began at estrous (18). However, there was an increase in the number of piglets weighing over 1.0 kg with tendencies in our study. These improvements align with previous studies indicating that sows supplemented with COS during gestation and lactation partly affected the litter performance (7, 19). Pervious study indicated that maternal COS supplementation during late gestation effectively enhanced placental nutrient transport capacity, improved angiogenesis within the placenta, alleviated placental oxidative stress and inflammatory response (20). Increased the number of piglets weighing over 1.0 kg may partly due to the facilitated placental function of COS.

Interestingly, maternal COS supplementation reduced weaning-to-estrus interval, consistent with prior research showing that mannan oligosaccharide added to late gestation sow diet shortened the interval (21). The weaning-to-estrus interval is a key component of non-productive days for sows, and shortened weaning-to-estrus interval has a potential to improve production efficiency on the next reproductive cycle in swine industry (22). The decreased weaning-to-estrus interval is associated with the concentration of serum reproductive hormones and the gut microbiota (23). Previous study has shown that COS increased the organ index of the ovary and serum estrogen level (24). The shortened weaning-to-estrus interval induced by COS in this study may be related to the reproductive hormones, further study was needed.

Our study revealed an increase in plasma fructose and TG concentrations in suckling piglets following maternal COS supplementation. Both fructose and TG are involved in lipid metabolic pathways, and fructose is a primary hexose sugar in fetal blood and amniotic fluids of piglet, playing a role in promoting growth and development during embryonic and fetal stages (25). Our current finding also demonstrated that COS supplementation increased the concentrations of protein, lactose, solids-not-fat, and TGF-β1 in milk, which was consistent with the previous study (14). The elevated milk components indicated that more energy and nutrients were available to the neonates, while the enhanced TGF-β1 likely provided effective immune protection to the offspring (7). The increased milk quality may in connection with the elevated porcine milk oligosaccharide diversity levels in sows supplemented with COS (18). The increases in plasma fructose and TG concentration as well as milk quality indicated that maternal COS intervention benefits stimulating growth and development of neonatal piglet, which was confirmed by increase in weaning weight and ADG of piglets. It is well known that the physiology status and performance of piglets depend on intestinal development and optimal functioning. Therefore, we subsequently investigated the effects of maternal dietary COS on intestinal development and health status in piglets.

During fetal, perinatal, neonatal, and postnatal periods, there are multiple critical growth and development windows of the intestine (2). The rapid growth of the small intestine occurs in the early suckling period, when the intestine must adapt to effectively digest and absorb nutrients and bioactive compounds presented in colostrum and milk (26). This growth is characterized by increased cell proliferation rate enhanced metabolic responses. In this study, COS supplementation in sow diet increased the mRNA expression of *HNF4A*, *SCDBP2*, and *MAP2K1* in jejunum of sucking piglets, and the *HNF4A* and *SCDBP2* had important roles in cell proliferation and differentiation (27, 28). More recently, it has been reported that *HNF4A* plays an important role in controlling claudin-7 expression during intestinal epithelial cell differentiation (29). This notion was further supported by our results that the expression of *OLDN3*, a member of the claudin family that plays a crucial role in controlling paracellular permeability in epithelia and endothelia (30), was enhanced in intestine of sucking piglets from sows supplemented with COS. Meanwhile, upregulated expression of gluconeogenesis gene (*G6PC* and *PCK1*), glycolysis-related gene (*LDHA*), as well as lipid biosynthesis gene (*SLC27A2*) was observed in intestine of sucking piglets from sows supplemented with COS. These findings were consistent with the previous study that COS could modulate metabolic profile such as fatty acid synthesis and decomposition, bile acid modification, and tryptophan metabolism (31). Overall, these observations supported that maternal COS supplementation could improve intestinal epithelial differentiation and glucose and lipid metabolism in suckling piglets.

Furthermore, COS is well recognized for its ability to enhance the immune response *in vivo* and *in vitro* (32). Our results showed that the gene expression of ileal *CD163* and *MHCII* of suckling piglets were increased by maternal dietary COS supplementation. Both *CD163* and *MHCII* are involved in the mononuclear phagocyte system, and their elevated levels indicate the orchestrated intestinal mucosal immune responses (33). In addition, the activation of *CD163* was generally accompanied by the changes of anti-inflammatory and pro-inflammatory mediators (34). This activation also correlated with an increased production of inflammatory cytokine IL-10 and INF-*γ* in ileum, indicating a protective role for COS in maintaining immune homeostasis. The changes in mucosal immune function observed in suckling piglets may be linked to the enhanced acquisition of passive immunity, as evidenced by direct effects on milk immunoglobulin levels in COS sows (35). Thus, our findings suggested that dietary intervention with COS in sows had beneficial effects on maintaining mucosal immune status in suckling piglets.

The weaning period is another critical window for the intestinal growth and development of piglets. Previous studies have shown that supplementation with COS can provide additional benefits to weaned piglets by improving intestinal nutrient digestion and absorption, enhancing intestinal histomorphology, promoting antioxidant capacity, and regulating immune response (36–38). In the present study, it was found that COS supplementation in sows led to increased intestinal density and enhanced intestinal morphology, particularly through decreased jejunal crypt dept. Histological changes such as villous atrophy and crypt elongation may increase susceptibility to intestinal infection after weaning, particularly in the distal part of small intestine which is more susceptible to inflammatory status (39). Enhanced intestinal morphology likely promotes greater immunoregulatory effects compared to post-weaning intervention alone, as evidenced by higher *CD163* mRNA expression and lower mRNA expression of *CCL2* and IL-10, INF-*γ* concentrations observed in the group receiving both maternal and post-weaning COS supplementation (COSm-COSp group). These results suggested that piglets who received COS from their mother’s milk during suckling period followed by solid feed during weaning exhibited improved intestinal mucosal structure and immunity. This enhanced effect on intestinal development may be attributed to the intake of nutrient substances and growth factors presented in milk of sows during suckling, although further research is needed to elucidate the underlying mechanisms.

Furthermore, the immediate weaning in piglets not only causes obviously structural and functional alterations in the small intestine but also contributes to an unstable microbiome in the hindgut (40). Therefore, we further investigated the impact of COS supplementation on gut microbiota in post-weaning piglets. Previous studies have shown that post-weaning piglets experience a decrease in *Lactobacillus* spp. and an increase in *Escherichia coli* due to sudden dietary and environmental changes (41). The abrupt increase of *Escherichia coli* after weaning has been linked to postweaning diarrhea in piglets (42). However, maternal COS intervention mitigated these disturbances in gut microbiota induced by weaning, consistent with the antimicrobial activity of chitosan against *Escherichia coli* infection in weaned piglets (43). Additionally, piglets in COSm-COSp group showed increased levels of SCFA such as acetate, propionate, and butyrate, which are metabolic byproducts of microorganisms. The SCFA with a total concentration of 50–200 mM can be efficiently absorbed by the gut mucosa (44). Acetate, as the main SCFA, serves as a substrate for cholesterol and fatty acid synthesis; propionate acts as a precursor for glucose synthesis; and butyrate is the primary energy source. All of them play important roles in regulating the development of gut epithelium (45).

## 5 Conclusion

The current study has shown that COS supplementation in sow’s diets during late gestation and lactation resulted in positive effects, including a reduction in the weaning to estrus interval, an increase the numbers of piglets weighing more than 1.0 kg, increased ADG of piglets during lactation, and improved milk composition. Moreover, maternal COS has shown beneficial effects on promoting intestinal proliferation and differentiation, regulating intestinal metabolism, and reducing intestinal inflammation in their offspring during suckling. Additionally, COS supplementation for both sows and piglets has been found to enhance intestinal growth and SCFA production while suppressing intestinal inflammation and reducing harmful bacteria content after weaning. These findings indicate that COS supplementation improves gut health during critical developmental windows from gestation through weaning.

## Data Availability

The original contributions presented in the study are included in the article and supplementary material, further inquiries can be directed to the corresponding author.

## References

[1] Bischoff S. 'Gut health': a new objective in medicine? BMC Med. (2011) 9:24. doi: 10.1186/1741-7015-9-24, PMID: 21401922 PMC3065426

[2] Meyer A Caton J. Role of the small intestine in developmental programming: impact of maternal nutrition on the dam and offspring. Adv Nutr. (2016) 7:169–78. doi: 10.3945/an.115.010405, PMID: 27180380 PMC4717893

[3] Meng Q Sun S Luo Z Shi B Shan A Cheng B. Maternal dietary resveratrol alleviates weaning-associated diarrhea and intestinal inflammation in pig offspring by changing intestinal gene expression and microbiota. Food Funct. (2019) 10:5626–43. doi: 10.1039/c9fo00637k, PMID: 31432838

[4] Langley-Evans SC. Nutrition in early life and the programming of adult disease: a review. J Hum Nutr Diet. (2015) 28:1–14. doi: 10.1111/jhn.12212, PMID: 24479490

[5] Thadathil N Velappan S. Recent developments in chitosanase research and its biotechnological applications: a review. Food Chem. (2014) 150:392–9. doi: 10.1016/j.foodchem.2013.10.083, PMID: 24360467

[6] Muanprasat C Chatsudthipong V. Chitosan oligosaccharide: biological activities and potential therapeutic applications. Pharmacol Ther. (2017) 170:80–97. doi: 10.1016/j.pharmthera.2016.10.013, PMID: 27773783

[7] Ho T Jahan M Haque Z Kracht S Wynn P Du Y. Maternal chitosan oligosaccharide intervention optimizes the production performance and health status of gilts and their offspring. Anim Nutr. (2020) 6:134–42. doi: 10.1016/j.aninu.2020.02.001, PMID: 32542193 PMC7283373

[8] Wan J Jiang F Zhang J Xu Q Chen D Yu B. Amniotic fluid metabolomics and biochemistry analysis provides novel insights into the diet-regulated foetal growth in a pig model. Sci Rep. (2017) 7:44782. doi: 10.1038/srep44782, PMID: 28300194 PMC5353717

[9] Xie C Wu X Long C Wang Q Fan Z Li S. Chitosan oligosaccharide affects antioxidant defense capacity and placental amino acids transport of sows. BMC Vet Res. (2016) 12:243. doi: 10.1186/s12917-016-0872-8, PMID: 27806719 PMC5094001

[10] Xie C Long X Yang H Fan Z Xiao D. Effect of maternal supplementation with chitosan oligosaccharide on the antioxidant capacity of suckling piglets. J Anim Sci. (2016) 94:453–6. doi: 10.2527/jas.2015-9611

[11] Yang C Ferket P Hong Q Zhou J Cao G Zhou L. Effect of chito-oligosaccharide on growth performance, intestinal barrier function, intestinal morphology and cecal microflora in weaned pigs. J Anim Sci. (2012) 90:2671–6. doi: 10.2527/jas.2011-4699, PMID: 22785166

[12] Liu P Piao X Kim S Wang L Shen Y Lee H. Effects of chito-oligosaccharide supplementation on the growth performance, nutrient digestibility, intestinal morphology, and fecal shedding of *Escherichia coli* and *Lactobacillus* in weaning pigs1. J Anim Sci. (2008) 86:2609–18. doi: 10.2527/jas.2007-0668, PMID: 18502883

[13] Wan J Jiang F Xu Q Chen D Yu B Huang Z. New insights into the role of chitosan oligosaccharide in enhancing growth performance, antioxidant capacity, immunity and intestinal development of weaned pigs. RSC Adv. (2017) 7:9669–79. doi: 10.1039/c7ra00142h

[14] Wan J Zhang J Chen D Yu B Mao X Zheng P. Alginate oligosaccharide-induced intestinal morphology, barrier function and epithelium apoptosis modifications have beneficial effects on the growth performance of weaned pigs. Ital J Anim Sci. (2018) 9:58. doi: 10.1186/s40104-018-0273-x, PMID: 30128148 PMC6094457

[15] Livak KJ Schmittgen TD. Analysis of relative gene expression data using real-time quantitative PCR and the 2^− ΔΔCT^ method. Methods. (2001) 25:402–8. doi: 10.1006/meth.2001.1262, PMID: 11846609

[16] Chen Y Mou D Hu L Zhen J Che L Fang Z. Effects of maternal low-energy diet during gestation on intestinal morphology, disaccharidase activity, and immune response to lipopolysaccharide challenge in pig offspring. Nutrients. (2017) 9:9. doi: 10.3390/nu9101115, PMID: 29027951 PMC5691731

[17] Edwards AK Wessels JM Kerr A Tayade C. An overview of molecular and cellular mechanisms associated with porcine pregnancy success or failure. Reprod Domest Anim. (2012) 47:394–401. doi: 10.1111/j.1439-0531.2012.02103.x, PMID: 22827397

[18] Cheng L Wang L Xu Q Huang L Zhou D Li Z. Chitooligosaccharide supplementation improves the reproductive performance and milk composition of sows. Livest Sci. (2015) 174:74–81. doi: 10.1016/j.livsci.2015.02.003

[19] Duan X Tian G Chen D Yang J Yu B. Effects of diet chitosan oligosaccharide on performance and immune response of sows and their offspring. Livest Sci. (2020) 239:104114. doi: 10.1016/j.livsci.2020.104114

[20] Wang X Fang T Chen D Pu J Tian G He J. Maternal chitosan oligosaccharide supplementation during late gestation and lactation optimizes placental function in sows and intestinal function in 21-day-old IUGR suckling piglets. Front Vet Sci. (2024) 11:1463707. doi: 10.3389/fvets.2024.1463707, PMID: 39606660 PMC11600973

[21] Duan X Chen D Zheng P Tian G Wang J Mao X. Effects of dietary mannan oligosaccharide supplementation on performance and immune response of sows and their offspring. Anim Feed Sci Technol. (2016) 218:17–25. doi: 10.1016/j.anifeedsci.2016.05.002

[22] Rempel LA Rohrer GA Nonneman DJ. Genomics and metabolomics of post-weaning return to estrus. Mol Reprod Dev. (2017) 84:987–93. doi: 10.1002/mrd.22820, PMID: 28436551

[23] Gu X Chen J Li H Song Z Chang L He X. Isomaltooligosaccharide and Bacillus regulate the duration of farrowing and weaning-estrous interval in sows during the perinatal period by changing the gut microbiota of sows. Anim Nutr. (2021) 7:72–83. doi: 10.1016/j.aninu.2020.06.010, PMID: 33997334 PMC8110870

[24] Huang Y Ye H Zhu F Hu C Zheng Y. The role of Chito-oligosaccharide in regulating ovarian germ stem cells function and restoring ovarian function in chemotherapy mice. Reprod Biol Endocrinol. (2021) 19:14. doi: 10.1186/s12958-021-00699-z, PMID: 33494759 PMC7830852

[25] Kim J Song G Wu G Bazer FW. Functional roles of fructose. Pro Natl Acad Sci. (2012) 109:E1619–28. doi: 10.1073/pnas.1204298109, PMID: 22623530 PMC3382482

[26] Xu RJ Mellor DJ Tungthanathanich P Birtles MJ Simpson HV. Growth and morphological changes in the small and the large intestine in piglets during the first three days after birth. J Dev Physiol. (1992) 18:161–72. PMID: 1284564

[27] Mortier E Wuytens G Leenaerts I Hannes F Heung MY Degeest G. Nuclear speckles and nucleoli targeting by PIP2-PDZ domain interactions. EMBO J. (2005) 24:2556–65. doi: 10.1038/sj.emboj.7600722, PMID: 15961997 PMC1176451

[28] Yeh M Bosch D Daoud S. Role of hepatocyte nuclear factor 4-alpha in gastrointestinal and liver diseases. World J Gastroenterol. (2019) 25:4074–91. doi: 10.3748/wjg.v25.i30.4074, PMID: 31435165 PMC6700705

[29] Farkas A Hilgarth R Capaldo C Gerner-Smidt C Powell D Vertino P. HNF4α regulates claudin-7 protein expression during intestinal epithelial differentiation. Am J Pathol. (2015) 185:2206–18. doi: 10.1016/j.ajpath.2015.04.023, PMID: 26216285 PMC4530128

[30] Garcia-Hernandez V Quiros M Nusrat A. Intestinal epithelial claudins: expression and regulation in homeostasis and inflammation. Ann N Y Acad Sci. (2017) 1397:66–79. doi: 10.1111/nyas.13360, PMID: 28493289 PMC5545801

[31] Ji X Zhu L Chang K Zhang R Chen Y Yin H. Chitooligosaccahrides: digestion characterization and effect of the degree of polymerization on gut microorganisms to manage the metabolome functional diversity in vitro. Carbohydr Polym. (2022) 275:118716. doi: 10.1016/j.carbpol.2021.118716, PMID: 34742440

[32] Naveed M Phil L Sohail M Hasnat M Baig MMFA Ihsan AU. Chitosan oligosaccharide (COS): an overview. Int J Biol Macromol. (2019) 129:827–43. doi: 10.1016/j.ijbiomac.2019.01.192, PMID: 30708011

[33] Bain C Mowat A. Macrophages in intestinal homeostasis and inflammation. Immunol Rev. (2014) 260:102–17. doi: 10.1111/imr.12192, PMID: 24942685 PMC4141699

[34] Van Gorp H Delputte P Nauwynck H. Scavenger receptor CD163, a Jack-of-all-trades and potential target for cell-directed therapy. Mol Immunol. (2010) 47:1650–60. doi: 10.1016/j.molimm.2010.02.008, PMID: 20299103

[35] Wan J Xu Q He J. Maternal chitosan oligosaccharide supplementation during late gestation and lactation affects offspring growth. Ital J Anim Sci. (2018) 17:994–1000. doi: 10.1080/1828051X.2018.1435313

[36] Suthongsa S Pichyangkura R Kalandakanond-Thongsong S Thongsong B. Effects of dietary levels of chito-oligosaccharide on ileal digestibility of nutrients, small intestinal morphology and crypt cell proliferation in weaned pigs. Livest Sci. (2017) 198:37–44. doi: 10.1016/j.livsci.2017.02.004

[37] Yang H Xiong X Li J Yin Y. Effects of chito-oligosaccharide on intestinal mucosal amino acid profiles and alkaline phosphatase activities, and serum biochemical variables in weaned piglets. Livest Sci. (2016) 190:141–6. doi: 10.1016/j.livsci.2016.06.008

[38] Xiong X Yang H Wang X Hu Q Liu CX Wu X. Effect of low dosage of chito-oligosaccharide supplementation on intestinal morphology, immune response, antioxidant capacity, and barrier function in weaned piglets. J Anim Sci. (2015) 93:1089–97. doi: 10.2527/jas.2014-7851, PMID: 26020885

[39] Wijtten PJ van der Meulen J Verstegen MW. Intestinal barrier function and absorption in pigs after weaning: a review. Br J Nutr. (2011) 105:967–81. doi: 10.1017/S0007114510005660, PMID: 21303573

[40] Pluske J Turpin D Kim J. Gastrointestinal tract (gut) health in the young pig. Anim Nutr. (2018) 4:187–96. doi: 10.1016/j.aninu.2017.12.004, PMID: 30140758 PMC6104527

[41] Gresse R Chaucheyras-Durand F Fleury MA Van de Wiele T Forano E Blanquet-Diot S. Gut microbiota dysbiosis in postweaning piglets: understanding the keys to health. Trends Microbiol. (2017) 25:851–73. doi: 10.1016/j.tim.2017.05.004, PMID: 28602521

[42] Tang F Wang J Li D Gao S Ren J Ma L. Comparative genomic analysis of 127 *Escherichia coli* strains isolated from domestic animals with diarrhea in China. BMC Genomics. (2019) 20:212. doi: 10.1186/s12864-019-5588-2, PMID: 30866824 PMC6416869

[43] Wan J Zhang J Wu G Chen D Yu B Huang Z. Amelioration of enterotoxigenic *Escherichia coli*-induced intestinal barrier disruption by low-molecular-weight chitosan in weaned pigs is related to suppressed intestinal inflammation and apoptosis. Int J Mol Sci. (2019) 20:3485. doi: 10.3390/ijms20143485, PMID: 31315208 PMC6678621

[44] Louis P Flint HJ. Formation of propionate and butyrate by the human colonic microbiota. Environ Microbiol. (2017) 19:29–41. doi: 10.1111/1462-2920.13589, PMID: 27928878

[45] Martin-Gallausiaux C Marinelli L Blottière HM Larraufie P Lapaque N. SCFA: mechanisms and functional importance in the gut. Proc Nutr Soc. (2021) 80:37–49. doi: 10.1017/s0029665120006916, PMID: 32238208

