# Supplementary material for: Effect of maternal and post-weaning dietary chitosan oligosaccharide supplementation on reproductive performance of sow and gut growth and development in offspring
Source: Front Vet Sci. 2025 Sep 23;12:1666462. doi: 10.3389/fvets.2025.1666462 (PMC12500443; doi:10.3389/fvets.2025.1666462)
Supplement: Supplementary file 1 [file Table_1.DOCX]

**Table S1** Primers sequences of target genes selected for analysis by real-time PCR.

| Names | Sequence of primers (5’---3’) | Accession No |
| --- | --- | --- |
| *CCL2* | F :CCTCATCCTCCAGCATGAAGGTCTCTGC | NM_214214.1 |
|  | R : GGTGGAGTCAGGCTTCAAGGCTTCGG |  |
| *CD163* | F : GATGTTCTGCCCATTTAAGTTCCT | NM_213976 |
|  | R : TGACCAAGCAGGCACTGAGA |  |
| *CLDN3* | F : ATCGGCAGCAGCATTATCAC | [FJ887981.1](https://www.ncbi.nlm.nih.gov/nuccore/227121292) |
|  | R : ACACTTTGCACTGCATCTGG |  |
| *CLDN7* | F : TATGAGTTTGGCCCTGCCATC | [FJ887979.1](https://www.ncbi.nlm.nih.gov/nuccore/227121288) |
|  | R : CCTTGGCAGAATTGGGCTTAG |  |
| *G6PC* | F : CGGCTTTCGGTGCTTGAA | EU295557.1 |
|  | R : CTGCACAGTCCAGAATCCCA |  |
| *HNF4A* | F : CTTCTTTGACCCAGATGCCAAG | DQ061106.1 |
|  | R : GGCGGTCGTTGATGTAATCCT |  |
| *IGF1R* | F : CAACCTCCGGCCTTTTACTTT | [U58370.1](https://www.ncbi.nlm.nih.gov/nucleotide/1378001?report=genbank&log$=nuclalign&blast_rank=4&RID=CDNZR8VU01N) |
|  | R : CAGGAATGTCATCTGCTCCTT |  |
| *LDHA* | F : ATCTTGACCTATGTGGCTTGGA | [FJ865398.1](https://www.ncbi.nlm.nih.gov/nuccore/268633766) |
|  | R : TCTTCAGGGAGACACCAGCAA |  |
| *MAP2K1* | F : TCGATTCCATGGCCAACTCC | NM_001143716.1 |
|  | R : ACCGCCATCTCAACCAGAGA |  |
| *MHCII* | F : AAGAAGGAGACTGTCTGGCG | NM _00114062.2 |
|  | R : GGAGCGTTTAGTCACGATGT |  |
| *mTOR* | F : ACAAGGACACAGCGACTCAG | XM_003127584 |
|  | R : CGCGGAACCAGTGAGGTAAT |  |
| *PCK1* | F : TCGAGAAAGCCTTCAATGCC | FJ668384.1 |
|  | R : GCGTGCGACCCTTCATG |  |
| *PPARGC1A* | F : CGCAAGCAATTTTTCAAGTCTAAC | NM_213963.1 |
|  | R : GGAAGCAGGATCAAAGTCATCTG |  |
| *SDCBP2* | F : CGGGCTGAAGGACAAAGAGG | NM_001244863.1 |
|  | R : GGGGATGATGGTCAAGGTGATG |  |
| *SLC27A2* | F : CTAAGAATACAGGACACCATTGAGA | NM_001278777.1 |
|  | R : AAGGCATCTTTGATAACCGCA |  |
| *TGFB2* | F : GGATCTTGGGTGGAAATGGA | XM_013978074.1 |
|  | R : GGCACAGAAGTTGGCATTGT |  |
| *HPRT1* | F : TACCTAATCATTATGCCGAGGATTT | [DQ845175.1](https://www.ncbi.nlm.nih.gov/nuccore/112980814) |
|  | R : AGCCGTTCAGTCCTGTCCAT |  |
| *PPIA* | F : AGCACTGGGGAGAAAGGATT | NM_214353 |
|  | R : AAAACTGGGAACCGTTTGTG |  |
| *TBP* | F : AACAGTTCAGTAGTTATGAGCCAGA | DQ845178.1 |
|  | R : AGATGTTCTCAAACGCTTCG |  |
